# Excellent response of intramedullary Erdheim-Chester disease to vemurafenib: a case report

**DOI:** 10.1186/s13104-015-1135-7

**Published:** 2015-04-30

**Authors:** Charalampos Tzoulis, Thomas Schwarzlmüller, Ivar Otto Gjerde, Eirik Søfteland, Gesche Neckelmann, Martin Biermann, Julien Haroche, Oddbjørn Straume, Olav Karsten Vintermyr

**Affiliations:** Department of Neurology, Haukeland University Hospital, Bergen, Norway; Department of Clinical Medicine, University of Bergen, Bergen, Norway; Department of Nuclear Medicine, Haukeland University Hospital, Bergen, Norway; Department of Medicine, Haukeland University Hospital, Bergen, Norway; Department of Radiology, Haukeland University Hospital, Bergen, Norway; Department of Internal Medicine & French reference center for rare auto-immune & systemic diseases, AP-HP. Pitié-Salpêtrière hospital, 47-83 bd de l’hôpital, 75013 Paris, France; Université Pierre et Marie Curie, UPMC Univ Paris 06, Paris, France; Department of Oncology, Haukeland University Hospital, Bergen, Norway; Department of Pathology, Haukeland University Hospital, Bergen, Norway; The Gade Laboratory for Pathology, Department of Clinical Medicine, University of Bergen, Bergen, Norway

**Keywords:** Erdheim Chester, Vemurafenib, BRAF, Treatment, Tumor, Histiocytosis, Spinal cord

## Abstract

**Background:**

Erdheim-Chester disease is a rare histiocytosis characterized by multi-systemic organ involvement. Immune-modulating agents such as interferon-alpha have limited success and the disorder is progressive and causes high morbidity and mortality. Treatment with the BRAF-inhibitor vemurafenib has recently produced substantial improvement in three patients with Erdheim-Chester disease expressing the p. V600E BRAF mutation. The disorder commonly affects the central nervous system and it is not yet known whether vemurafenib can reverse intra-axial infiltration and the resulting neurological impairment.

**Case presentation:**

In this work, we report for the first time an excellent clinical response to vemurafenib in a Norwegian patient with debilitating progressive spastic paraparesis due to intra-axial infiltration of the thoracic spinal cord. The patient had been unresponsive to interferon-alpha. Low dose vemurafenib (720 mg daily) for a period of three months resulted in significant tumor shrinkage by >60% and regression of contrast enhancement and fluorodeoxyglucose uptake on positron-emission tomography. The patient’s spastic paraparesis and gait function improved dramatically. She currently walks unaided and reports a substantially improved quality of life.

**Conclusion:**

Our findings show that vemurafenib therapy, even at low doses, can be effective for the treatment of intra-axial central nervous system involvement in BRAF-positive Erdheim-Chester disease.

## Background

Erdheim-Chester disease (ECD) is a rare form of non-Langerhans histiocytosis characterized by widespread, multi-systemic infiltration by histiocytes that express CD68, but not CD1a or S-100 protein [1]. Commonly affected organs include the skeleton, skin, thoracic and abdominal internal organs and the central nervous system (CNS). In the nervous system, ECD commonly affects the neurohypophysis causing diabetes insipidus. Approximately a third of the cases with CNS disease show extrahypophyseal involvement comprising intra-axial infiltrative lesions, meningioma-like masses and periarterial infiltration [1-3]. Spinal cord involvement may also occur due to either extramedullary masses or intra-axial infiltration [1,4-6].

Interferon-alpha, which is commonly used as first-line therapy, has limited effectivity and chronic use may be complicated by severe side effects [7]. Various second-line agents have been used including anakinra [8], cladribine [9], infliximab [10] and tyrosine kinase inhibitors [11], but systematic data are lacking and the effectivity of these therapies remains unknown. ECD has an invariably progressive course and is associated with high morbidity. Mortality rate was found to be 22% in one large study [1,12-14].

It was recently shown that approximately 54% of the patients with ECD and 57% of patients with Langerhans cell histiocytosis harbor a somatic gain-of-function mutation (p. V600E) in the protooncogene *BRAF*. This mutation leads to activation of the tumorigenic RAS-ERK pathway and is thought to play a key role in the pathogenesis of the disorder [15]. A trial of vemurafenib, a specific inhibitor of mutant BRAF resulted in substantial clinical improvement in three patients with ECD and Langerhans cell histiocytosis [16] offering a novel and effective therapeutic option for these severe disorders.

We report an excellent clinical response to vemurafenib in an ECD patient with spastic paraparesis secondary to an intramedullary infiltrative lesion. This is the first report of vemurafenib response in ECD with intra-axial CNS infiltration. Our findings show that vemurafenib therapy is effective for the treatment of infiltrative CNS lesions in BRAF-positive ECD.

## Case presentation

A now 34 year old female of Norwegian origin was referred to us at the age of 31 with a 10 year history of progressive spastic paraparesis due to an intramedullary tumor. Her history and clinical features at the time of diagnosis have been described elsewhere in detail [17]. MRI of the head and spine showed diffuse cerebellar leukoencephalopathy, multiple vertebral changes with a hypointense appearance on T1 and T2 sequences and an intramedullary tumor in the spinal cord extending from the 4th-6th thoracic vertebrae. Scintigraphy with technetium 99 (Tc^99^) and fluorodeoxyglucose positron-emission tomography (FDG-PET) showed extensive tracer uptake in the epiphyseal long bones, spine and skull and hypermetabolism in the cord tumor. She also had hypophyseal involvement with central diabetes insipidus and hypogonadotropic hypogonadism. Bone marrow biopsy of a vertebral lesion was performed to confirm the diagnosis of ECD.

The patient was given interferon-alpha for a period of three years during which her intramedullary tumor gradually expanded accompanied by progressive worsening of her spastic paraplegia. At the time of this study (baseline evaluation) she could walk short distances with the help of two crutches, was severely unsteady and had experienced several falls (Table 1).

**Table 1:** **Clinical and functional measures before and during therapy with vemurafenib**

**Measure**	**Baseline**	**1 month**	**3 months**
Tumor size (mm^2^)	142	65	58.8
Spasticity knee (Ashworth scale)	2	2	1
Spasticity ankle (Ashworth scale)	1	1	0
Walking aids	two crutches	variable	none
Gait test*	300	370 m	405 m
Legg press	30 kg	not done	70 kg
Treadmill	1 km	1-2 km	3 km

Tumor size is given in area of the largest cross section through the tumor.

*Maximal distance at fast walking pace during 6 minutes.

**Figure 1:**
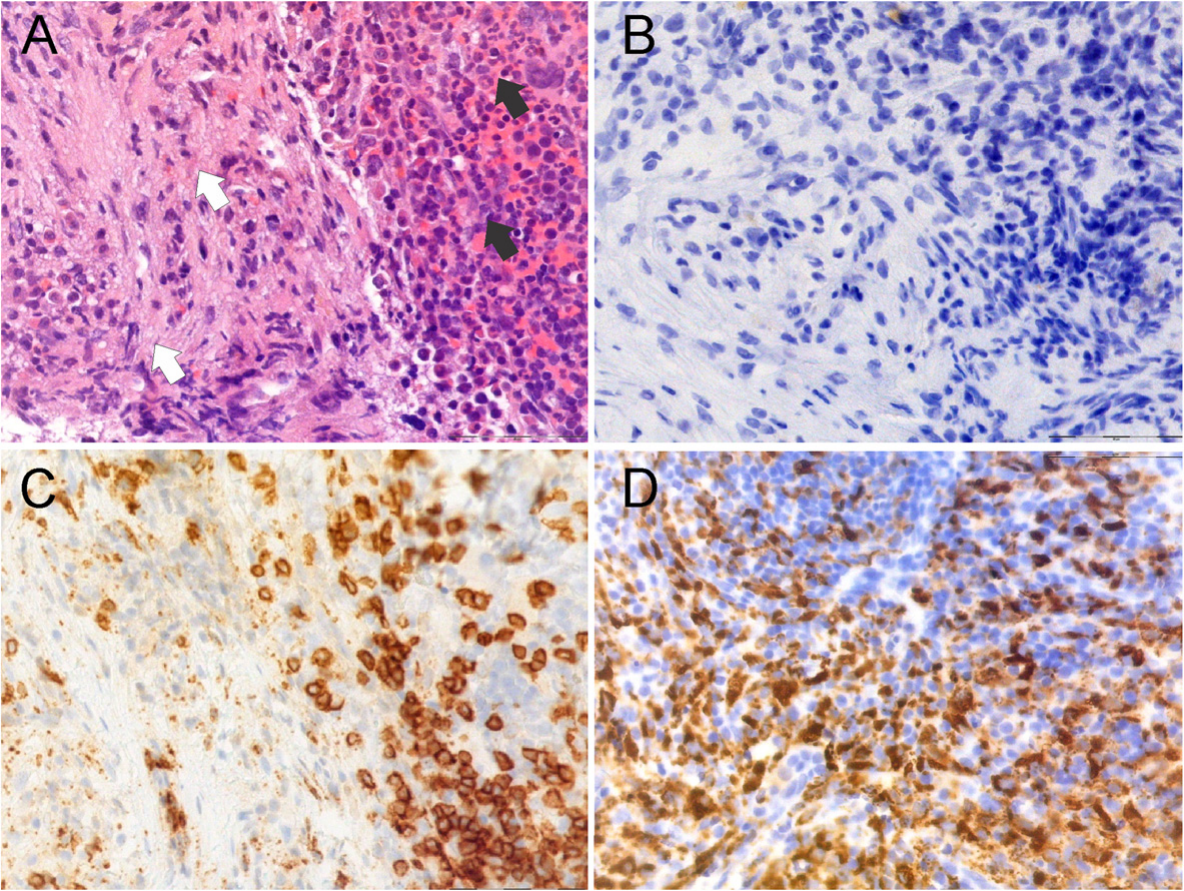
Pathology. Serial sections of the patient’s vertebral trephine biopsy showing normal (black arrows) and affected (white arrows) bone marrow. Sections have been stained with hematoxylin-eosin **(A)**, CD1a **(B)**, CD3 **(C)** and CD68 **(D)**.

In order to determine the patient’s BRAF status, her vertebral biopsy was stained with appropriate markers and severely affected areas identified, dissected and tested for mutations. The biopsy was stained with hematoxylin-eosin and immunohistochemically with monoclonal mouse anti-human CD68, clone PGM1, diluted 1:100, polyclonal rabbit anti-human CD3, diluted 1:400, and monoclonal mouse anti-human CD1a, diluted 1:500 (all from DAKO Glostrup, Denmark) (Figure 1).

The most severely affected area of the biopsy was identified and macroscopically dissected. The dissected material was treated in deparaffinization solution (Qiagen, Hilden, Germany), extracted in ATL lysis buffer (Qiagen, Hilden, Germany) and digested overnight with proteinase K (Qiagen, Hilden, Germany) at 56°C, as previously described [18]. DNA was extracted using the DSP DNA mini kit (Qiagen, Hilden, Germany) on an automated (QIAsymphony) platform (Qiagen, Hilden Germany). *BRAF* mutations were analysed by real time PCR (Rotor-Gene Q) using an allele-specific mutation detection kit from Qiagen (Therasceen BRAF RGQ, Manchester, UK) according to the manufacturer’s protocol. The biopsy material tested positive for the common BRAF p. V600E mutation.

The patient was started with vemurafenib 960 mg daily (480 mg bid). Treatment response was monitored at baseline (one week before treatment), and at one and three months after treatment start. At each point the patient was evaluated by MRI of the CNS, whole-body FDG-PET and clinical assessment including Ashworth spasticity grading. Gait function and lower limb strength were assessed by treadmill, weight lifting by leg-press and a standardized gait-test testing maximal distance at fast walking pace during a six minute period.

In addition, she underwent thorough cardiological and dermatological examination. Routine blood count and chemistry and electrocardiography were performed monthly.

After the first week of treatment with vemurafenib the patient developed side-effects in the form of severe skeletal pain and joint rubor and swelling. Side-effects were dose-dependent and subsided completely when the dose was reduced to 720 mg daily (240 mg + 480 mg), which she currently uses. Vemurafenib treatment resulted in significant clinical and functional improvement, which was already evident by the end of the first month and is ongoing. The clinical and functional measures of the patient’s condition are summarized in Table 1.

**Figure 2:**
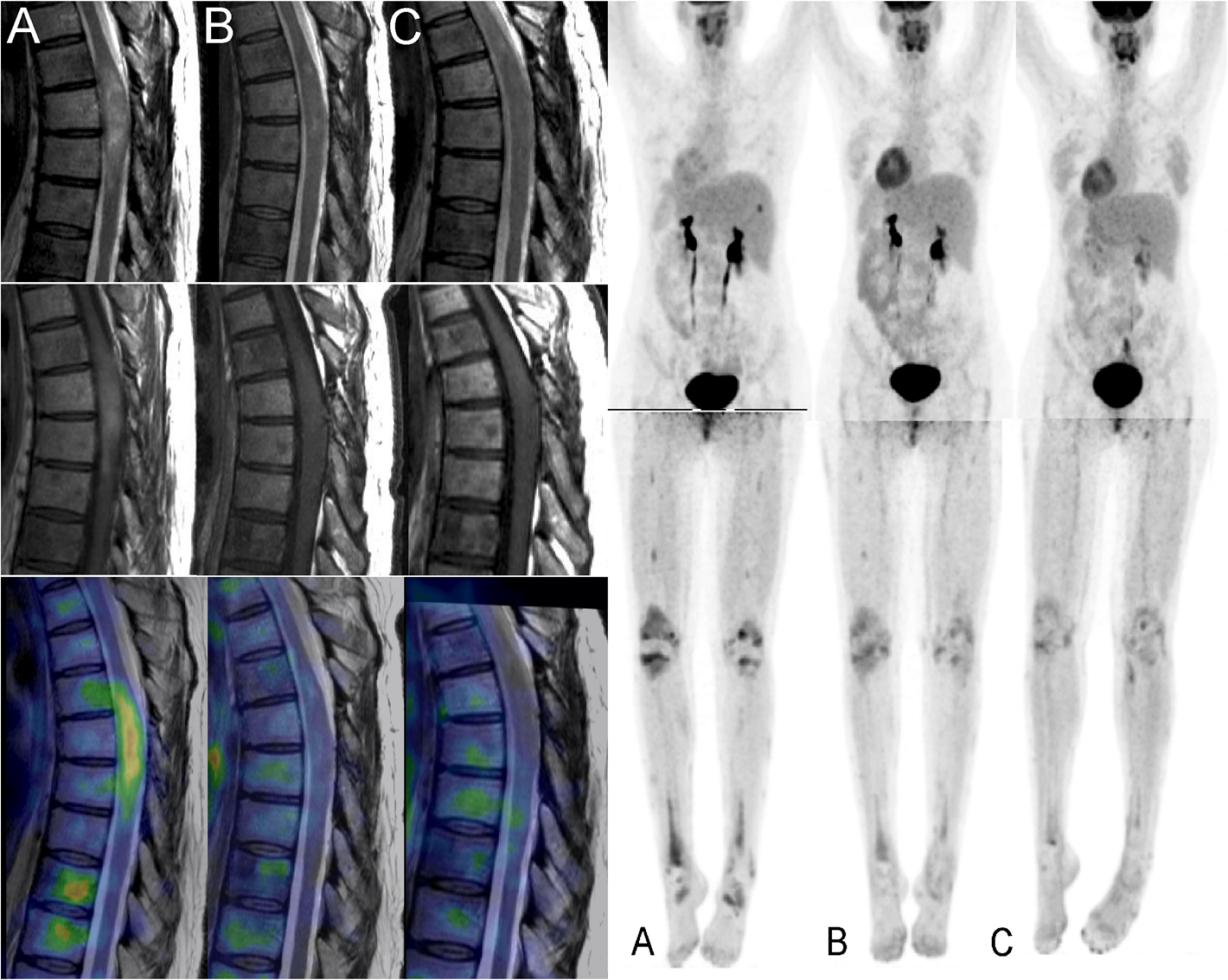
Treatment response. Left panel: Sagittal imaging of the spinal cord tumor at the level of the 2nd-7th thoracic vertebrae by T2-weighted MRI (upper row), T1-weighted, gadolinium enhanced MRI (middle row) and fusion with FDG-PET (lower row) taken at baseline **(A)**, one month **(B)** and three months **(C)** of vemurafenib therapy. There is gradual regression of the intramedullary tumor’s size, edema (T2 signal) and contrast-enhancement. PET shows complete disappearance of FDG uptake in the tumor and substantial reduction in the bodies of the 6th and 7th thoracic vertebrae. Right panel: whole body FDG-PET taken at the same time points showing gradual regression of hot-spots in the patient’s skeleton and in particular the lower limbs.

The intramedullary lesion regressed in size by >60% to nearly normal cord thickness, consistent with partial remission according to Response Evaluation Criteria In Solid Tumors (RECIST) criteria. Also, the lesion showed normalization of T2 signal and loss of contrast enhancement suggesting restoration of the local blood–brain barrier. FDG-PET showed substantial reduction of tracer uptake in the cord lesion and skeletal changes (Figure 2). The patient’s spastic paraparesis and gait function improved significantly during the first three months of treatment (Table 1). She stopped using the crutches and currently walks unaided and reports a substantial increase in activities of daily function as well as improved quality of life.

She is now in the fourth month of treatment and tolerates the current dose well with only mild side-effects in the form of intermittent, mild rubor and pain in the small joints of the hands and feet and moderate skin photosensitivity necessitating the use of sun-screen.

## Conclusions

We show that vemurafenib is an effective and well tolerated therapy for the treatment of intra-axial CNS infiltrative lesions in ECD patients who are positive for the p. V600E *BRAF* mutation, suggesting a neoplastic origin of the disease. In spite of long-standing neurological impairment, our patient showed substantial functional improvement, which correlated well with the reduction in lesion size and activity. This suggests that neurological dysfunction in ECD patients can have favorable functional outcome with therapy irrespective of symptom or disease duration.

Interestingly, a daily dose of 720 mg was sufficient to elicit and maintain an ongoing positive clinical response in our patient. This dose is substantially lower than the recommended dose in metastatic malignant melanoma (1920 mg daily) and even lower than the dose used in the three reported ECD patients (960 mg daily) [16]. An important challenge in treating ECD patients with vemurafenib is that they require long-term treatment with a compound which has substantial dose-dependent toxicity. Progression free survival for metastatic melanoma treated with vemurafenib is 6.9 months and median follow up time in the BRIM-3 trial was 12.5 months [19]. Response duration in ECD patients remains to be determined in clinical trials and long term side effects, including secondary malignancies, need to be carefully monitored. Our findings suggest that the minimum-effective dose for ECD may be substantially lower than for other indications. This should be considered in ongoing and future clinical trials and low-dose regimes should be tested in order to determine the minimum-effective dose for this group of patients.

### Consent

Written informed consent was obtained from the patient for publication of this Case Report and any accompanying images. A copy of the written consent is available for review by the Editor-in-Chief of this journal.

## Abbreviations

CNS: Central nervous system
ECD: Erdheim Chester Disease
FDG-PET: Fluorodeoxyglucose positron-emission tomography
MRI: Magnetic resonance imaging
CT: Computed tomography
RECIST: Response evaluation criteria in solid tumors
